# Hypothalamic Orexin Projections to the Hippocampal CA1 Region Alleviate Cognitive and Synaptic Plasticity Impairments Induced by Blue Light Exposure

**DOI:** 10.1111/cns.70551

**Published:** 2025-08-17

**Authors:** Zhe Feng, Qingqing Li, Zhenquan He, Baocong Yu, Ting Mi, Jiandong Niu, Yuhong He, Qi Li, Xi Chen, Jianguo Niu, Dan Ding

**Affiliations:** ^1^ School of Basic Medical Sciences Ningxia Medical University Yinchuan China; ^2^ Key Laboratory of Craniocerebral Diseases of Ningxia Hui Autonomous Region Ningxia Medical University Yinchuan China; ^3^ Ningxia Regional Characteristic Traditional Chinese Medicine Collaborative Innovation Center Ningxia Medical University Yinchuan China; ^4^ Department of Radiology General Hospital of Ningxia Medical University Yinchuan China; ^5^ School of Clinical Medicine Sciences Ningxia Medical University Yinchuan China

**Keywords:** blue light exposure, cognitive impairment, neural pathway of hypothalamic orexin neurons to hippocampus CA1, synaptic plasticity

## Abstract

**Background and Objectives:**

Exposure to blue light emitted from electronic devices has been shown to impair cognitive performance; however, the mechanisms underlying these deleterious effects remain poorly understood. Orexin neurons in the hypothalamus, which play a key role in modulating cognitive processes and synaptic plasticity, project directly to the hippocampus, a brain region critical for learning and memory. Therefore, our study provides novel insights into the neural mechanisms underlying blue light‐related cognitive dysfunction.

**Methods and Results:**

In this study, we evaluated cognitive impairments in mice subjected to 21 days of blue light exposure using open‐field, novel object recognition, and Morris water maze tests. Electrophysiological recordings and Golgi staining revealed that synaptic plasticity was significantly impaired in blue light‐exposed mice. The results of biochemical experiments indicated that the expression of Orexin‐A, along with the synaptic plasticity‐related factors PSD‐95 and SYN‐1, was downregulated at both the protein and gene levels in the hippocampus of mice following blue light exposure. Furthermore, retrograde tracing combined with immunofluorescence staining showed that hypothalamic orexin neurons projected to the hippocampus, and that CTb‐labeled orexin neurons were significantly activated in the hypothalamus (c‐FOS^+^) of blue light‐exposed mice. Notably, we found that chemogenetic activation of the hypothalamic orexin–hippocampus neural pathway significantly alleviated cognitive functions, accompanied by enhanced expression of Orexin‐A, PSD‐95, and SYN‐1 at both the protein and gene levels.

**Conclusions:**

These findings suggest that the hypothalamic orexin projections to the hippocampal CA1 region alleviate cognitive and synaptic plasticity impairments induced by blue light exposure.

## Graphical abstract

The neural pathways from hypothalamic orexin neurons to the hippocampus improve the impairment of learning and memory induced by blue light exposure, revealing the mechanism of improved damage caused by blue light.

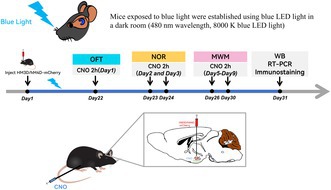

## 1 Introduction

With the widespread adoption of electronic devices, children are increasingly surrounded by digital screens in their daily environments. Prolonged screen exposure during early childhood has emerged as a global concern, and the escalating trend of early‐age exposure is particularly alarming [1, 2]. The period between ages 3 and 6, commonly referred to as the preschool years, is a pivotal phase in the cognitive development of children [3]. During this critical timeframe, exposure to electronic screens can trigger a cascade of adverse effects on the learning, memory, and overall brain development of preschool‐aged children [4].

Among the various types of radiation emitted by electronic screens, blue light is considered the most harmful. With a wavelength range of 400–480 nm, blue light carries comparatively high energy [5]. The adverse effects of blue light include impairments in learning, memory, and cognition [6]. Research indicates that children, who are in a crucial phase of neurological growth, are particularly vulnerable to the adverse effects of blue light. Children are significantly more vulnerable to the harmful effects of blue light exposure compared to adults, due to their developing eyes and higher light transmittance to the retina [7]. Prolonged exposure to blue light emitted from screens significantly hinders children's learning abilities and brain development [8]. Therefore, elucidating the mechanisms underlying blue light‐induced cognitive damage is of urgent importance.

Blue light is actively involved in regulating a wide range of brain functions [9]. Studies suggest that blue light is detected by the retina and transmitted via intrinsically photosensitive retinal ganglion cells, which project to several brain regions, including the hypothalamus, indicating that the hypothalamus may be involved in mediating the effects of blue light [10]. Neuronal synaptic plasticity forms the neurobiological foundation for the growth and development of the nervous system, as well as for neuronal damage and repair, cognitive behavior, learning, and memory. This encompasses alterations in the number, morphology, and function of neurites and synapses [11, 12]. The hippocampus has been extensively studied and recognized as a critical brain region responsible for advanced neural functions, such as learning and memory [13, 14]. It directly shapes the processes of learning and memory and concurrently plays an essential role in the development of cognitive functions [15]. Moreover, Synapsin I (SYN‐1), located on the presynaptic membrane, is vital for normal neuronal development through its interactions with presynaptic proteins and the actin cytoskeleton [16]. Additionally, postsynaptic density‐95 (PSD‐95) is integral to the formation and maintenance of synaptic connections among neurons in the central nervous system, thereby maintaining neuronal homeostasis [17, 18].

Orexin, also known as hypocretin, is a neuropeptide released by a group of neurons situated in the posterior/lateral hypothalamus (LH) [19]. The orexin system plays a significant role in cognitive functions such as learning and memory [20]. A Previous study has demonstrated that orexin can influence spatial learning and memory by altering structural and functional connections within the hippocampus [21]. Orexin‐A induces hippocampal synaptic plasticity by modulating the acetylcholine, glutamate, gamma‐aminobutyric acid (GABA), and adrenergic systems, influencing hippocampal learning and memory [22]. Moreover, Orexin‐A enhances the excitatory activity of neurons in the hippocampus CA1 region, thereby enhancing learning and memory capabilities. Accumulating evidence has validated the participation of hypothalamic orexin neurons in regulating cognitive functions associated with learning and memory, exerting a notable impact on their plasticity [23]. However, whether orexin signaling via the hypothalamic–hippocampal pathway can alleviate the conditions of blue light‐induced impairments in learning and memory remains unclear.

Building upon these findings, we hypothesize that projections from hypothalamic orexin neurons to the hippocampus may alleviate blue light‐induced impairments in cognition and synaptic plasticity. To test this hypothesis, we conducted a comprehensive set of experiments: (i) a series of behavioral experiments were performed after 21 days of blue light exposure to assess changes in cognitive functions; (ii) synaptic plasticity deficits were assessed using Golgi staining and patch‐clamp electrophysiology; (iii) Western blotting and RT–qPCR were employed to confirm the activation of synaptic plasticity‐related factors downstream; (iv) retrograde tracing techniques and immunofluorescence staining (IF) were used to identify the hypothalamic orexin‐hippocampus neural pathway and examine the activation of orexin neurons in mice following blue light exposure; (v) finally, chemogenetics were used to modulate this pathway and examine its effects. The aim of this study is to elucidate the neural mechanisms through which blue light impairs cognitive functions and to explore the potential of hypothalamic orexin pathways as therapeutic targets for restoring learning and memory capacities disrupted by blue light exposure.

## 2 Materials and Methods

### 2.1 Animals and Experimental Groups

All experiments were conducted using male C57BL/6J wild‐type (WT) mice and Hcrt‐IRES‐Cre (orexin‐Cre) mice, aged 3–4 weeks, to eliminate variability due to hormonal fluctuations in females. All mice were housed under controlled conditions: temperature (22°C ± 1°C), humidity (40%–60%), and a 12/12‐h light/dark cycle (lights on at 7:00 and off at 19:00), with ad libitum access to food and water. WT mice were purchased from the Laboratory Animal Centre of Ningxia Medical University (Yinchuan, China), and Hcrt‐IRES‐Cre mice were obtained from Shanghai Model Organisms Center Inc. All procedures complied with governmental regulations on the use of laboratory animals. The approval was obtained from the Committee on Ethics and Welfare of Laboratory Animals, Ningxia Medical University (Grant No. 2023A0506).

After 1 week of environmental adaptation, the mice were randomly assigned to two groups and housed in cabinets under different light–dark cycles for 21 days. One group was maintained under a standard white light–dark cycle (12:12; light ~150 lx/dark 0 lx, “White”), while the other group was exposed to a dim blue light–dark cycle (12:12; dim blue light ~5 lx/dark 0 lx, “Blue”). Blue light exposure was achieved using blue LED lights (480 nm wavelength, 8000 K) in a dark room, whereas white light exposure was provided from 7:00 to 19:00 each day. Dim light conditions were created by placing blue LED lights above the racks holding the mouse cages. The intensity and consistency of the lighting were measured using a lux meter. Throughout the experimental period, body weight, food intake, and water consumption were monitored regularly. Three‐week‐old mice (*n* = 12) were exposed to blue light for 21 days, and follow‐up experiments were subsequently performed.

### 2.2 Behavioral Tests

The Open‐field test (OFT), Novel object‐recognition test (NORT) and Morris water‐maze test (MWT) were used to assess the impact of blue light exposure on the cognition of young mice.

### 2.3 Open‐Field Test (OFT)

The OFT was used to quantify exploratory behavior and assess locomotor parameters, such as total distance traveled and time spent moving. Additionally, it served to habituate the animals to the test apparatus prior to the novel object recognition test (NORT), which was conducted on the following day, thereby shortening the overall experimental timeline. For the OFT, each mouse was placed in the center of an open‐field arena enclosed by walls measuring 50 cm × 50 cm × 40 cm (length × width × height). The apparatus was situated in a sound‐isolated experimental room illuminated with diffuse lighting. On the test day, the mouse was placed at the center of the arena, and its locomotor activity was recorded for 5 min using a video camera positioned above the field and tracked with Smart 3.0 software. At the end of each session, the total distance traveled was calculated. To prevent olfactory interference, the arena was thoroughly cleaned with 75% alcohol between trials.

### 2.4 Novel Object Recognition Test (NORT)

The NORT is a behavioral paradigm designed to assess learning and memory in animals by leveraging their innate tendency to explore novel objects and their natural habituation to familiar objects. The test was carried out similarly to the procedure previously described for the OFT apparatus; however, the OFT apparatus was modified to include two objects for exploration. After each trial, the objects used in the experiment and the testing box itself were disinfected with a 75% ethanol solution. The test consisted of three phases: habituation (achieved through the OFT), familiarization, and choice. Each animal was tested individually. During the familiarization phase, the animals were placed in the area where two identical objects, marked A1 and A2, were placed. The animals were allowed to explore the apparatus freely for 5 min. On the next day, one of the objects was replaced with another novel object (object B). The new and familiar objects are presented in different colors, structures, and shapes. Object exploration was classified as the mouse placing its head at a distance of 2 cm from any object. During the experiment, the time spent exploring individual objects during each phase and the total time spent exploring both objects were measured. The recognition index (RI), the time spent researching the new object relative to the total exploration of the objects [RI = tB/(tB + tA1)], was determined.

### 2.5 Morris Water‐Maze Test (MWT)

The MWT includes a place navigation test and a spatial probe trial, which was used to detect spatial learning and memory [24]. The place navigation test, conducted over five consecutive days, evaluates spatial learning. During the first 4 days, the mice underwent training, and the formal test was performed on the 5th day. Each day, the mice were released into the water from the middle of the four quadrants of the maze, with their backs facing the wall of the box, and the time to find the platform was considered escape latency. If a mouse failed to locate the platform within 60 s, it was gently guided to the platform, where it was allowed to remain for 15 s, and the escape latency was recorded as 60 s.

The spatial probe trial was used to detect spatial memory. The underwater platform was removed on the 5th day, the quadrant farthest from the underwater platform was removed, and the number of mice that passed through the platform within 60 s was recorded.

### 2.6 Golgi Staining

Three mice, randomly selected from each group (White and Blue), were rapidly decapitated following deep anesthesia, and their brains were immediately extracted for processing. The FD Rapid Golgi Stain Kit, which includes solutions A, B, C, D, and E, along with the required consumables, was used for the experiment. Fresh brain tissues were immersed in a mixture of A liquid and B liquid for 2 weeks, followed by immersion in Solution C for 3 days. Subsequently, 100‐μm‐thick sections were prepared from the tissues using a vibratome (Leica, Wetzlar, Germany). The sections were washed with double‐distilled water for 8 min and then dehydrated using 50%, 75%, 95%, and 100% ethanol for 16 min each. Finally, the slides were sealed with neutral gum, and Sholl analysis was performed with ImageJ software (Bethesda, MD, USA) to analyze and visualize the tissues.

### 2.7 Real‐Time Fluorescence Quantitative Polymerase Chain Reaction (RT–qPCR)

Total RNA was isolated from hippocampal tissues using an RNA kit (TIANGEN, Beijing, China). The concentration and purity of the RNA were assessed using a NanoDrop2000C spectrophotometer (Thermo Fisher Scientific, Waltham, MA, USA). Subsequently, the mRNA was reverse transcribed into complementary DNA using a reverse transcription kit (TaKaRa, Shiga, Japan) following the manufacturer's instructions. The RT–qPCR was conducted using SYBR Green to quantify the mRNA expression levels of PSD‐95 and SYN‐1, with Gapdh serving as the internal reference gene. All primers were synthesized by Shanghai Sangon Biotech Co. Ltd. (Shanghai, China). The primer sequences were as follows:

- *Psd 95*: sense, 5′‐GGAACCAAGGCGGATCGTGATC‐3′; antisense: 5′‐CCCAGCAAGGATGAAGGAGATGAAG‐3′
- *Syn1*: sense, 5′‐CATTCTGGGATGGGCAAGGCTAAG‐3′; antisense, 5′‐GGCTCAGCAGTGGCATATGTCTTAG‐3′
- *Gapdh*: sense, 5′‐GGTTGTCTCCTGCGACTTCA‐3′; antisense, 5′‐TGGTCCAGGGTTTCTTACTCC‐3′

The PCR was performed on a CFX‐96 Real‐Time PCR System (Bio‐Rad, Hercules, CA, USA) under the following cycling conditions: 40 cycles at 95°C for 10 s and 61.5°C for 30 s. The Ct values of the samples were recorded, and relative gene expression levels were calculated using the international relative quantitation method ($2^{-∆∆C_{t}}$). The expression levels of the target genes were expressed relative to the internal reference gne.

### 2.8 Western Blotting

Three mice from each group were anesthetized with isoflurane and decapitated, and their hippocampus was rapidly removed for analysis. The separated tissues were extracted using protein lysis buffer, and the protein concentration was determined using a BCA assay kit (Beyotime Biotechnology, China). The total protein (40 μg) from each sample was separated via 10% sodium dodecyl sulfate‐polyacrylamide gel electrophoresis (SDS‐PAGE) and subsequently transferred onto a 0.22 μm polyvinylidene fluoride (PVDF) membrane. Membranes were blocked using a protein‐free fast‐blocking solution and then incubated overnight at 4°C with the following primary antibodies: anti‐PSD‐95 (1:2000, AF5283, Affinity Biosciences, Cincinnati, USA), anti‐SYN‐1 (1:2000, AF6201, Affinity Biosciences, Cincinnati, USA), and anti‐GAPDH (1:5000, AP0063, Biogot, China). After five washes (5 min each), membranes were incubated with the IRDye 800CW goat anti‐rabbit IgG (H&L) secondary antibody (1:5000; LI‐COR Biosciences, Lincoln, NE, USA) for 2 h at room temperature. Protein expression was visualized using an Odyssey Infrared Imaging System (CLx‐0796, LI‐COR Biosciences).

### 2.9 Enzyme‐Linked Immunosorbent Assay (ELISA)

An enzyme‐linked immunosorbent assay (ELISA) was used to investigate the variations in Orexin‐A (OXA) content within the hippocampus via a mouse OXA assay kit (Nine‐Biotech Co. Ltd.). Hippocampal tissue specimens were retrieved, cut, and weighed (1 g of tissue), after which approximately 9 mL of PBS (pH 7.2–7.4) was added. The specimens were homogenized using manual or mechanical tools. After centrifugation at 2000–3000 rpm for 20 min, the supernatant was collected carefully. A portion was used for testing, while the remainder was stored at low temperatures. If precipitation occurred during storage, centrifugation was repeated.

The necessary strips were extracted from the aluminum foil bag after equilibrating to room temperature for 20 min, and the remaining strips were securely sealed in a self‐sealing bag and returned to 4°C. Standard and sample wells were prepared, with varying concentrations of the standard added to each standard well (50 μL). For the test sample wells, 10 μL of the test sample was added first, followed by 40 μL of sample diluent. Subsequently, 100 μL of horseradish peroxidase (HRP)‐labeled detection antibody was added to each well in both the standard and sample groups. The reaction wells were sealed with a membrane, and the plates were incubated for 60 min at 37°C in a water bath or a constant temperature incubator. After incubation, the liquid was discarded, and the wells were dried by gently patting on absorbent paper. The wells were filled with wash solution and left to stand for 1 min, after which the wash solution was removed. This washing process was repeated five times (an automatic plate washer could also be used). Subsequently, 50 μL of substrates A and B were added to each well, and the plates were incubated at 37°C in the dark for 15 min. Finally, 50 μL of stop solution was added, and the optical density (OD) values of each well were determined at a wavelength of 450 nm within 15 min. Utilizing the OD values of the measured standards as the *x*‐axis and the concentrations of the standards as the *y*‐axis, a standard curve was constructed on graph paper or through relevant software such as ELISACalc. The linear regression equation was derived, and the OD values of the samples were substituted into the equation to calculate the concentration of the samples.

### 2.10 Patch‐Clamp Electrophysiological Tests

Patch clamp electrophysiological tests were performed to study synaptic plasticity in acute hippocampal slices obtained from 6‐week‐old mice. After decapitation and brain extraction, the hippocampus was isolated, placed in ice‐cold sucrose‐based cutting solution (in mM: KCl 2.5, NaH_2_PO_4_ 1.25, NaHCO_3_ 25, d‐glucose 10, sucrose 210, Na‐ascorbate 1.3, CaCl_2_ 0.5, and MgSO_4_ 7), and sliced into 350 μm coronal sections using a vibratome (Leica, VT1000S). The slices were then incubated in artificial cerebrospinal fluid (ACSF; mM: NaCl 119, KCl 2.5, NaHCO_3_ 26.2, NaH_2_PO_4_ 1, d‐glucose 11, CaCl_2_‐2H_2_O 2.5, MgSO_4_ 1.3, glucose 11, saturated with 95% O_2_/5% CO_2_) at 34°C for 15 min and then at room temperature for 60 min prior to electrophysiological recording. The slices were perfused with ACSF containing PTX and saturated with 95% O_2_/5% CO_2_ at a flow rate of approximately 3 mL/min during recording. The recording pipettes were filled with ACSF and had a resistance of 3–5 MΩ. Stimulation of Schaffer collaterals (SCs) was introduced using a concentric bipolar electrode placed in the stratum radiatum of the CA1 region. Field long‐term potentiation (LTP) was induced by a theta burst (TBS), consisting of six episodes at intervals of 10 s. Each episode of TBS comprised five bursts at 5 Hz, with each burst composed of five pulses at 100 Hz. Field excitatory postsynaptic potential (fEPSP) recordings were conducted 20 min at baseline and 60 min after induction. The fEPSP slope was analyzed to measure the field LTP.

Data acquisition was performed using Patchmaster software (Heka Elektronic Gmbh, Lambrecht/Pfalz, Germany), sampled at 10 kHz, and low‐pass filtered at 2 kHz. Offline data analysis was carried out using Fitmaster (Heka Elektronik Gmbh) or Clampfit10 (Molecular Devices, San Jose, CA, USA).

### 2.11 Stereotaxic Surgery

To determine the neural pathway from hypothalamic orexin neurons to the hippocampus, surgery was performed on the mice (*n* = 5). Cholera toxin b subunit (CTb) was injected into the hippocampus under microscopic guidance.

One week later, the mice were sacrificed, and coronal brain sections containing the hypothalamus and hippocampus were collected for immunofluorescence staining to visualize CTb and orexin to determine the pathway from orexin neurons in the hypothalamus to the hippocampus. WT mice were anesthetized with isoflurane (1.0%–1.5%, RWD Life Science, Shenzhen, China), and the skull was positioned horizontally and fixed on a stereotactic instrument (RWD Life Science). After the skin was incised to expose the skull, a 1–2 mm hole was drilled, and CTb (0.2%, 30 nL, List Labs, Campbell, CA, USA) was injected into the unilateral hippocampal region (coordinates: Bregma AP = −0.218 mm, ML = +1.000 mm, DV = −0.125 mm).

To observe the changes in learning, memory, and synaptic plasticity induced by chemogenetics activation or inhibition of the hypothalamic orexin–hippocampus pathway in blue light‐exposed mice, Hcrt‐IRES‐Cre mice were used. After anesthesia, CTb was injected into the hippocampus as described above. The rAAV‐Ef1a‐DIO‐hM3D (Gq)‐mCherry‐WREs viral construct (AAV2/9, > 2.00E+12 vg/mL; provided by Brain VTA, Wuhan, China) was injected into the bilateral hypothalamus of Hcrt‐IRES‐Cre mice (*n* = 6) at a volume of 200 nL per side (Bregma: AP = −1.300 mm, ML = ±0.700 mm, DV = −5.000 mm). Mice were then subjected to 21 days of blue light exposure. After this period, clozapine N‐oxide (CNO) was injected into the abdominal cavity of the mice (10 g/0.1 mL) to activate upstream orexin neurons. Similarly, the rAAV‐Ef1a‐DIO‐hM4D (Gi)‐mCherry‐WREs (AAV2/9, > 2.00E+12 vg/mL; also provided by Brain VTA) was injected into the bilateral hypothalamus of a separate group of Hcrt‐IRES‐Cre mice (*n* = 6), following the same procedure described above. Clozapine N‐oxide (CNO) was administered intraperitoneally after 21 days of blue light exposure.

### 2.12 Brain Tissue Preparation and Immunofluorescent Staining

Mice were deeply anesthetized with isoflurane and transcardially perfused with 200 mL of 0.9% saline, followed by 50 mL of 4% paraformaldehyde. The brain tissues were then removed, postfixed in 4% paraformaldehyde for 8 h, dehydrated in 30% sucrose for 48 h, and subsequently cut into 35 μm coronal sections using a cryostat (Leica).

Double immunofluorescence staining for CTb and orexin was used to study the orexin–hippocampus pathway in the hypothalamus. Brain tissue sections containing hypothalamic and hippocampal regions were incubated overnight with goat anti‐CTb (1:1000, Lot# 7032A9, List Labs) and rabbit anti‐orexin‐A (1:1000, ABCAM, Cambridge, MA, USA) at 4°C in 0.3% Triton X‐100. The sections were then rinsed three times with phosphate‐buffered saline (PBS) and incubated with CYTM3‐labeled donkey anti‐goat IgG (1:500, Lot# 134527, ABCAM) and Alexa Fluor 488‐labeled donkey anti‐rabbit IgG (1:500, batch number 136422, ABCAM). The membranes were then incubated with the secondary antibody for 3 h at room temperature. The brain sections were washed three times with PBS, mounted on slides, and covered with a fluorescent sealant.

The effects of blue light on the activation of orexin‐expressing neurons in the hypothalamus were studied using double‐immunofluorescence staining for orexin and c‐FOS. Hypothalamus‐containing brain tissue sections were incubated overnight with rabbit anti‐orexin‐A (1:1000, ABCAM) and rat anti‐c‐FOS (1:1000, Cat# 226017, Synaptic Systems, Coventry, UK) at 4°C in 0.3% Triton X‐100. They were then washed three times with PBS and incubated with Alexa Fluor 488‐labeled donkey anti‐rabbit IgG (1:500, Lot# 136422, ABCAM) and CYTM5‐labeled donkey anti‐rat IgG (1:500, Lot# 154628, ABCAM) secondary antibodies for 3 h at room temperature. The sections were subsequently washed three times with PBS, placed on slides, covered with a fluorescent sealant, and observed under a Las X microscope (Leica).

### 2.13 Data Quantification and Statistical Analysis

Following the exclusion of two mice due to inaccurate CTb injection sites, the number of CTb‐labeled soma and orexin^+^ co‐labeled cells at each site of the hypothalamus was manually quantified and recorded using the ImageJ counting tool. Six representative brain sections from each mouse were counted, and the number of orexin^+^ and c‐FOS^+^ co‐labeled cells in the hypothalamus was determined. Additionally, the number of mCherry^+^ and orexin^+^ co‐labeled cells in the hypothalamic slices was calculated for three representative brain sections per mouse. All cell counts were conducted manually in ImageJ.

Statistical analyses were performed using GraphPad Prism software (version 8.0.1; GraphPad Inc., La Jolla, CA, USA), and data are expressed as the mean ± standard error of the mean (SEM). All statistical analyses were two‐tailed. Comparisons between two groups were made using either paired or unpaired Student's *t*‐tests, depending on the experimental design. For analyses involving more than two groups, one‐way or two‐way analysis of variance (ANOVA) was employed, followed by Tukey's or Bonferroni's post hoc tests where appropriate. *p* < 0.05 was considered statistically significant. Statistical significance is indicated in the figures as follows: *p < 0.05, **p < 0.01, ***p < 0.001.

## 3 Results

### 3.1 Exposure to Blue Light Reduced Exploratory Behavior and Impaired Learning and Memory in Mice

**FIGURE 1:**
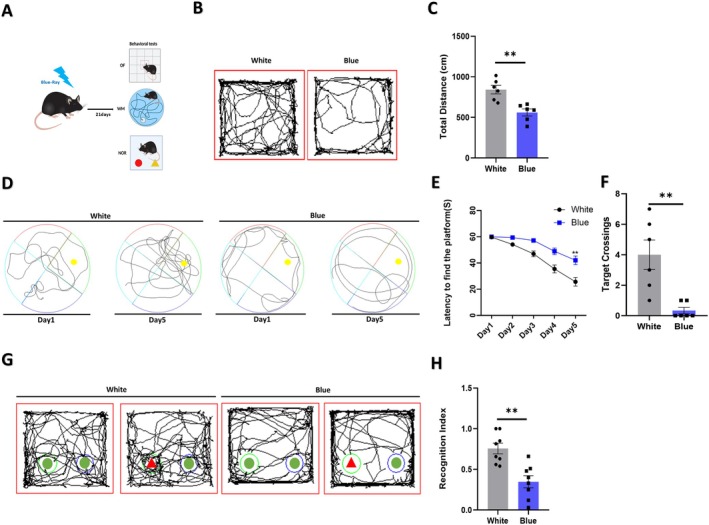
The cognitive function of mice is impaired after blue light exposure. (A) Schematic of the behavioral experiments performed on mice after 21 days of blue light exposure. (B) Trajectory chart of the open‐field test (*n* = 6 mice per group). (C) The mice traveled a shorter total distance in the open‐field test following blue light exposure (*p* = 0.021). (D) Trajectory of the Morris water maze (*n* = 6 mice per group). (E, F) The mice showed an increased latency to find the platform (*p* = 0.0055) and a decreased number of passes through the target platform (*p* = 0.004) in the Morris water maze following blue light exposure. (G) Trajectory chart of the novel object‐recognition test (*n* = 6 mice per group). (H) The mice spent less time on the novel object after blue light exposure (*p* = 0.001). The data are presented as the mean ± standard errors of the means. **p < 0.01. *T*‐tests were performed.

To assess the impact of blue light exposure on the learning and memory of mice, behavioral experiments were conducted. The results revealed that, following 21 days of exposure to blue light (Figure 1A), the autonomous motor skills and exploratory behavior in novel environments of mice were diminished. Additionally, there was a decrease in spatial positioning and orientation (spatial orientation) learning and memory abilities. The detailed findings are outlined as follows:

#### 3.1.1 Open‐Field Test Results

Compared to those in the control group (white light‐exposed mice), the total distance traveled in the open‐field area was significantly lower in the blue light‐exposed group (*n* = 6, 28.09% ± 6.83%, *p* = 0.021) (Figure 1B,C). The results indicate that autonomous movement and exploration of new environments decreased in young mice after 21 days of blue light exposure, further explaining the observed cognitive impairment.

#### 3.1.2 Morris Water Maze Test Results

The latency to find the platform increased in the blue light group compared to the control group (*n* = 6, 16.42% ± 4.66%, *p* = 0.0055) (Figure 1D,E). The number of platform crossings in the target quadrant was also lower in blue light‐exposed mice (*n* = 6, 3.67% ± 0.99%; *p* = 0.004) (Figure 1D–F), indicating that spatial learning and memory were impaired in mice after 21 days of blue light exposure.

#### 3.1.3 Novel Object Recognition Test Results

The findings from this segment revealed that the recognition index (RI) was significantly lower in the blue light‐exposed mice than in the white light‐exposed mice (*n* = 6, 0.41% ± 0.10%, *p* = 0.001) (Figure 1G,H). This indicated a reduction in exploratory interest toward novel objects and a decline in memory performance in the blue light‐exposed group.

Taken together, these results suggested that prolonged exposure to blue light impairs cognitive behaviors in mice.

### 3.2 Exposure to Blue Light Reduced Dendrite Complexity and Spine Density and Impaired Synaptic Plasticity in the Hippocampus of Mice

**FIGURE 2:**
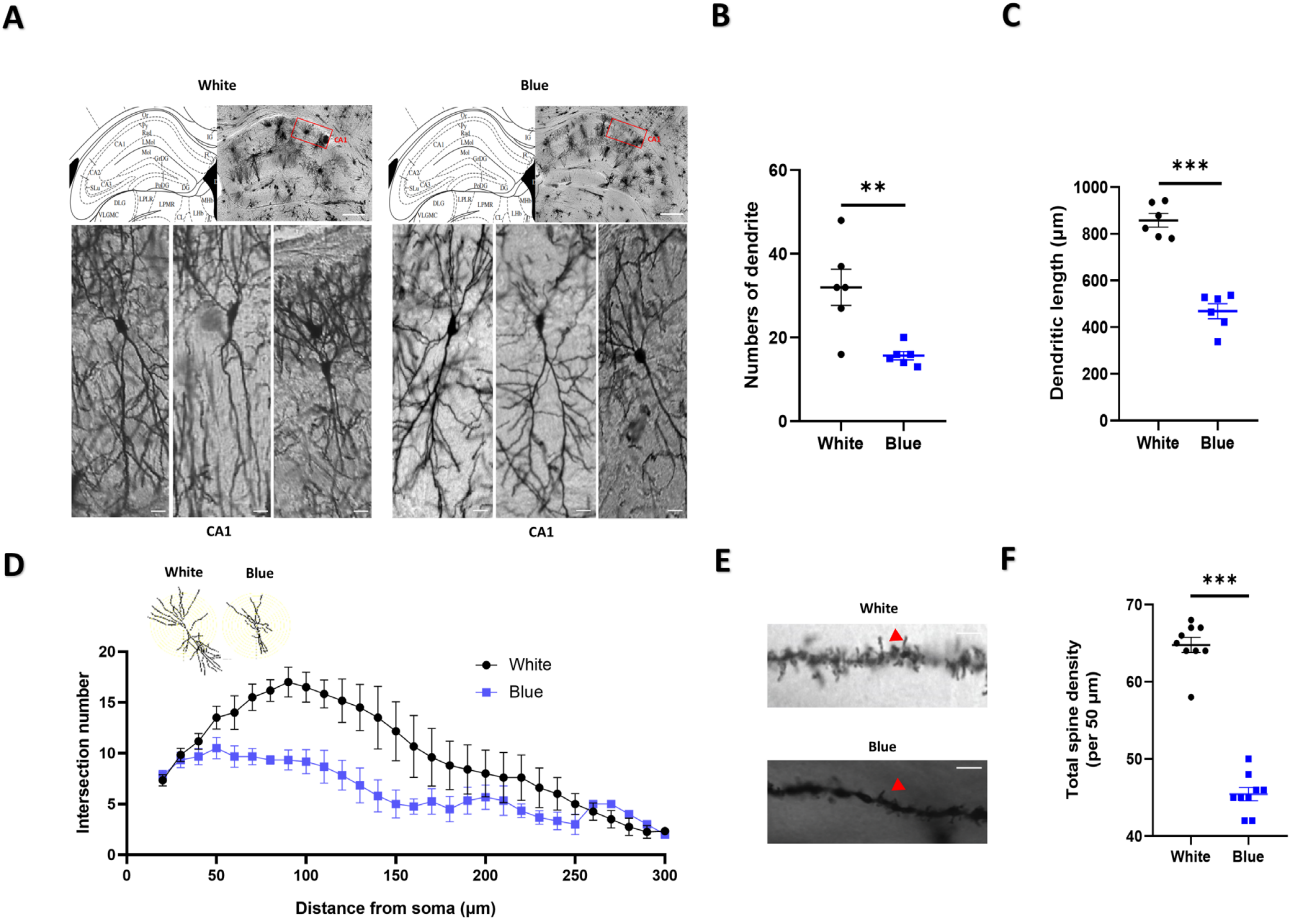
The complexity and spine density of neuron dendrites are decreased in the hippocampus of mice after blue light exposure. (A) Golgi staining of neurons and a schematic diagram of the neurons (*n* = 3 mice per group). Scale bar: 300 μm (hippocampus), 30 μm (CA1). (B, C) The number (*p* = 0.0043) and length (*p* = 0.0000123) of dendrites in the CA1 region were decreased in mice following blue light exposure (*n* = 6). (D) Sholl analysis of CA1 regions showing that dendritic complexities were decreased in mice following blue light exposure (*n* = 6). (E) Golgi staining of dendritic spines. The red arrow points to the spine. Scale bar: 5 μm. (F) The total number of dendritic spines in the CA1 region was decreased in mice following blue light exposure (*n* = 9) (*p* = 0.0000107). The data are presented as the mean ± standard errors of the means; **p < 0.01, ***p < 0.001. *T*‐tests were performed.

We proceeded to investigate the potential impact of blue light exposure on dendrite complexity and spine density through Golgi staining (Figure 2A). The results revealed a decrease in the dendrite complexity of neurons within the CA1 region (Figure 2D). Further analysis demonstrated that the blue light‐exposed mice displayed a reduction in the number of dendrites (*n* = 6, 16.33% ± 4.44%, *p* = 0.0043) (Figure 2B) and dendrite length (*n* = 6, 389.40% ± 43.08%, *p* = 0.0000123) (Figure 2C) in the CA1 regions. Furthermore, a reduction in total spine density was observed in the CA1 area of the blue light‐exposed mice (*n* = 9, 1.21% ± 20.67%, *p* = 0.0000107) (Figure 2E,F). The collective data strongly indicated that exposure to blue light results in decreased dendrite complexity and total dendritic spine density in mice.

**FIGURE 3:**
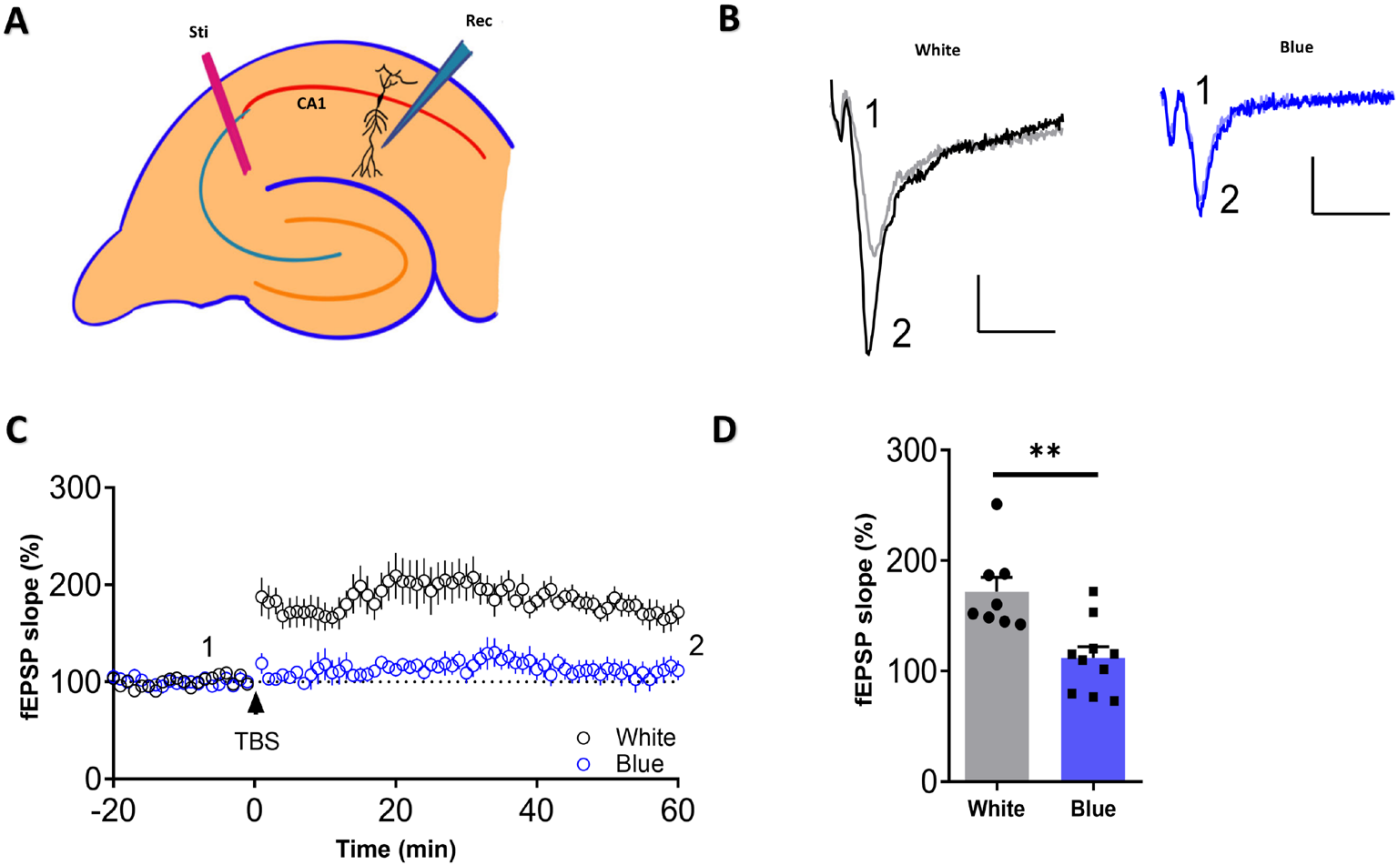
Synaptic plasticity is impaired in mice after blue light exposure. (A) Schematic diagram of the experimental material. Sti (Stimulation): The position of the stimulating electrode; Rec (Recording): Mark the position of the recording electrode. (B) The sample traces are the field excitatory postsynaptic potential (fEPSP) before TBS (1) or 60 min after LTP induction (2) Scale bar: 10 ms, 0.5 mV. (C) Hippocampal slices from mice exposed to blue light were significantly impaired in TBS‐induced LTP. White, *n* = 8; blue, *n* = 10. (D) The bar graph shows the percentage of the baseline at 60 min after TBS stimulation (*p* = 0.002). White, *n* = 8; blue, *n* = 10. The data represent the mean ± standard errors of the means; **p < 0.01. *T*‐tests were performed.

To corroborate the impact of blue light exposure on synaptic plasticity, we recorded the field LTP response of Schaffer collaterals SC‐CA1 synapses. Field LTP induced by theta burst stimulation (TBS) (Figure 3A) was impaired in mice after blue light exposure (Figure 3B–D) (*n* = 8, 60.02% ± 16.26%, *p* = 0.002), indicating that the blue light exposure impaired the synaptic plasticity of mice.

### 3.3 Exposure to Blue Light Decreased the Expression of Hippocampal Orexin‐A and the Synaptic Plasticity‐Related Factors PSD‐95 and SYN‐1 at Both the Protein and Gene Levels in Mice

**FIGURE 4:**
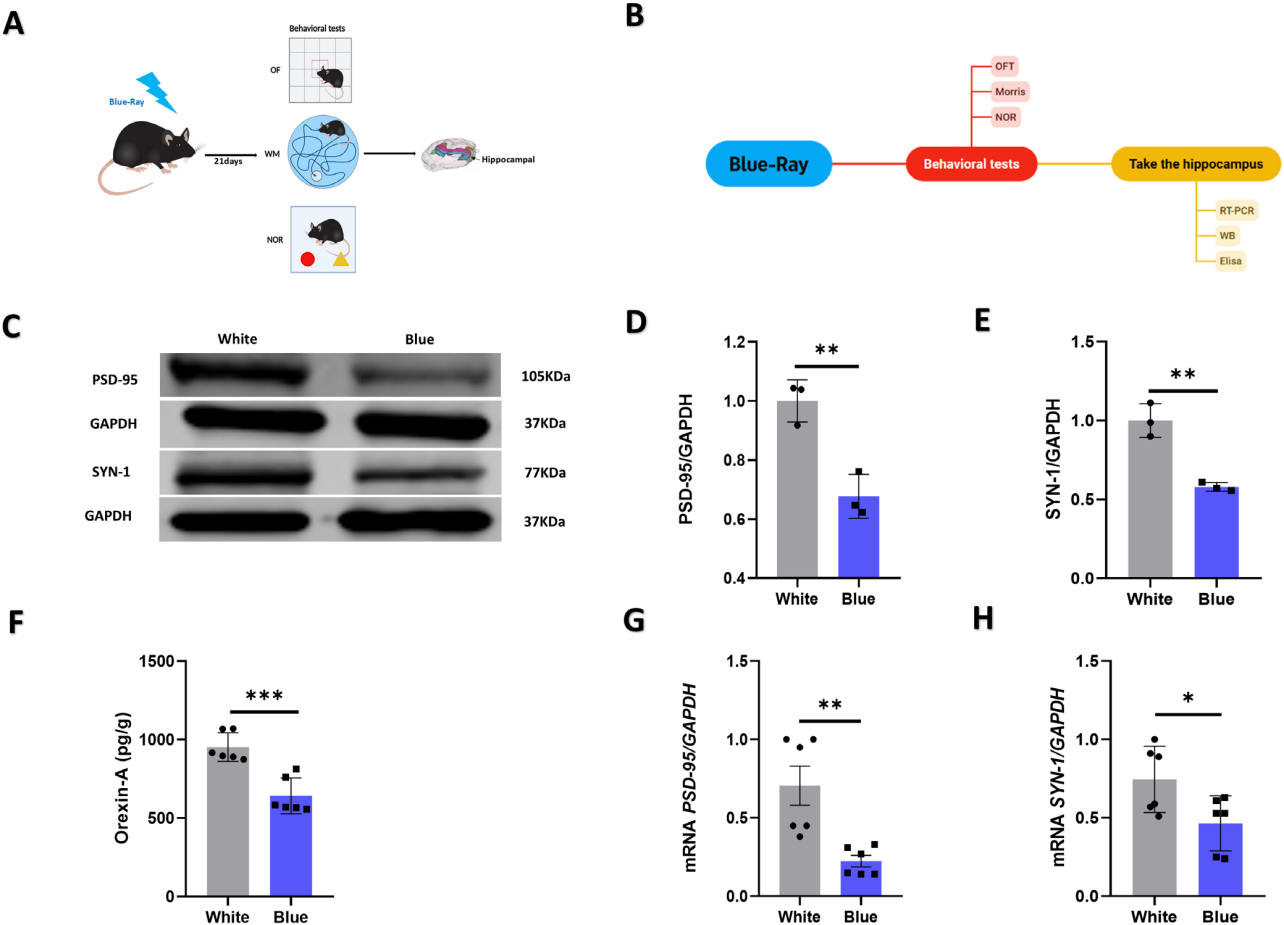
The expression of Orexin‐A, PSD‐95, and SYN‐1 were downregulated in the hippocampus of mice after blue light exposure. (A, B) Schematic diagram and time course of the experiment involving mice after 21 days of blue light exposure. (C) Western blotting analysis revealed the total expression of PSD‐95 and SYN‐1 in the hippocampus. (D) Quantitative analysis showed that the protein expression level of PSD‐95 was decreased in mice following blue light exposure (*p* = 0.006). White, *n* = 3; blue, *n* = 3. (E) Quantitative analysis showed that the protein expression level of SYN‐1 was decreased in mice following blue light exposure (*p* = 0.003). White, *n* = 3; blue, *n* = 3. (F) Quantitative analysis showed that the expression level of orexin‐A was decreased in mice following blue light exposure (*p* = 0.0004). White, *n* = 6; blue, *n* = 6. (G) Quantitative analysis showed that the mRNA level of PSD‐95 was decreased in the hippocampi of mice following blue light exposure (*p* = 0.0041). White, *n* = 6; blue, *n* = 6. (H) Quantitative analysis showed that the mRNA level of SYN‐1 was decreased in the hippocampus of mice following blue light exposure (*p* = 0.032). White, *n* = 6; blue, *n* = 6. The data are presented as the mean ± standard errors of the means; *p < 0.05, **p < 0.01, ***p < 0.001. *T*‐tests were performed.

The normal expression of synaptic plasticity factors is critical for maintaining both synaptic function and cognitive performance. Therefore, we detected their protein expression levels in the hippocampus by Western blotting. We found the expression levels of synaptic plasticity‐related proteins, specifically PSD‐95 (*n* = 3, 0.06% ± 0.32%, *p* = 0.006) (Figure 4C,D), and SYN‐1 (*n* = 3, 0.06% ± 0.42%, *p* = 0.003) (Figure 4C,E) were downregulated in the hippocampus compared to the white light‐exposed group. Furthermore, hippocampal Orexin‐A levels were also markedly reduced following blue light exposure (*n* = 6, 310.9% ± 59.62%, *p* = 0.0004) (Figure 4F). Additional details are provided in Table S1.

We further assessed the mRNA expression levels of *Psd‐95* and *Syn‐1* in the hippocampus through RT‐qPCR. The data showed the mRNA expression of *Psd‐95* (*n* = 6, 0.13% ± 0.48%, *p* = 0.0041) (Figure 4G) and *Syn‐1* (*n* = 6, 0.11% ± 0.28%, *p* = 0.032) (Figure 4H) were also decreased in the hippocampus of blue light–exposed mice.

### 3.4 Exposure to Blue Light Activated the Orexin‐Hippocampus Pathway

**FIGURE 5:**
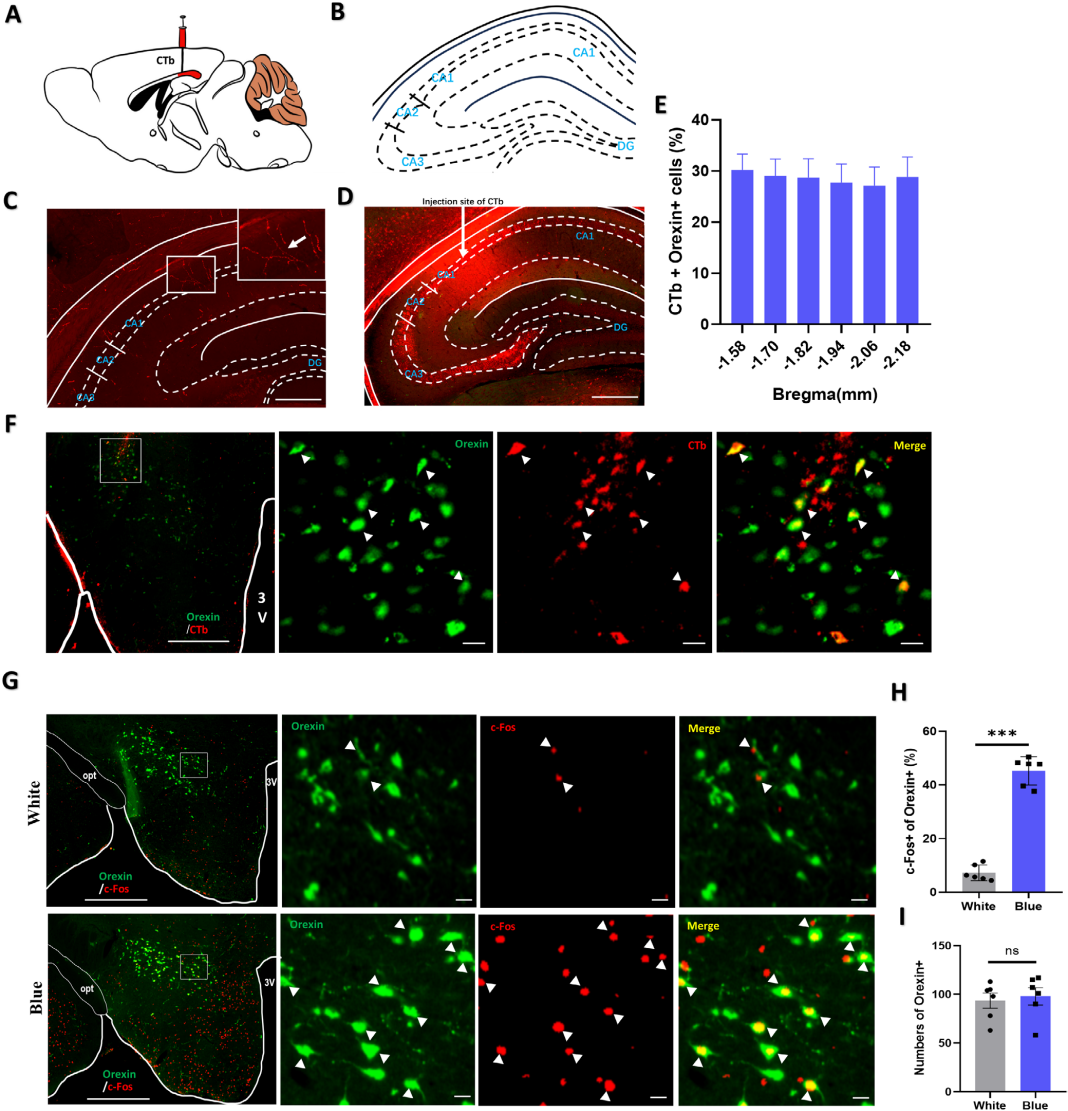
Hypothalamic orexin neurons are activated after blue light exposure. (A, B) Schematic diagram of the site at which CTb was injected into the hippocampal CA1 region of mice. (C) Photographs showing the presence of orexin immunofluorescence positive axonal fibers and terminals in the hippocampus in mice. The arrow indicates the axonal fibers and terminals of the orexin neurons in the CA1 region of hippocampus (*n* = 3 mice per group). Scale bar: 1000 μm. (D) Photograph showing the performance of injecting the retrograde tracer CTb into the hippocampus and conducting immunofluorescence staining in mice. The arrow indicates the center of the CTb injection site within the CA1 region of the hippocampus, which contains a large number of orexin axons. Scale bar: 1000 μm. (E) Quantification of the percentage of CTb^+^ and orexin^+^ colocalization in all orexin^+^ neurons in each hypothalamus section (*n* = 3 mice per group). (F) Images of CTb (red) and orexin (green) immunofluorescence staining in the hypothalamus, with squared areas enlarged in the images on the right. 3 V, 3rd ventricle. Scale bar: Left image 500 μm, right image 100 μm. (G) Images of immunofluorescence staining for orexin (green) and c‐FOS (red) in the hypothalamus of white and blue light‐exposed mice, with squared areas enlarged in the images on the right. 3 V, 3rd ventricle; opt, optic tract. Scale bars: Left images 500 μm, right images 100 μm. (H) The percentage of orexin neurons that were activated (c‐FOS^+^) in white light‐exposed mice and blue light‐exposed mice (*n* = 3 mice per group, *p* = 0.00000256). (I) The number of Orexin+ neurons in white light‐exposed mice and blue light‐exposed mice (*n* = 3 mice per group). The data represent the mean ± standard errors of the means; ***p < 0.001, ns: Nonsignificant. *T*‐tests were performed.

Orexins regulate a wide variety of biological functions, with a prominent role in cognitive processes [25]. To investigate whether blue light affects orexin neuronal activity, we examined the activation of orexin neurons in the hypothalamus using immunofluorescence staining. Orexin^+^ neurons and c‐FOS^+^ cells were observed in the hypothalamus (Figure 5G), with a subset of orexin^+^ neurons also positive for c‐FOS. The percentage of double‐stained neurons was significantly increased in mice after blue light exposure (*n* = 6, white vs. blue: 7.23% ± 2.46% vs. 45.26% ± 1.98%, *p* = 0.00000256) (Figure 5H). In addition, the number of orexin^+^ neurons in the hypothalamus did not differ significantly between the groups (Figure 5I), suggesting that the blue light exposure activates the orexin neurons in the hypothalamus.

Given that previous study has reported that orexin neurons project to the hippocampus [26], we examined brain tissue sections from young wild‐type mice. The analysis revealed the presence of orexin axon fibers and expanded axon terminals in the hippocampus (Figure 5C), confirming projections from hypothalamic orexin neurons to the hippocampus.

To further validate this pathway, a retrograde tracing experiment was conducted. CTb was injected into the CA1 region of the hippocampus, where orexin axon fibers and terminals were distributed abundantly in wild‐type mice (Figure 5A–D). Subsequently, double immunofluorescence staining for CTb and orexin in hypothalamic coronal sections was performed. Numerous CTb‐labeled cell bodies were observed, and some of which were also orexin positive^+^ (Figure 5F). It was calculated that 28.62% ± 1.11% of the CTb‐labeled cell bodies were immunopositive for orexin neurons (Figure 5E). This result strongly supports the projection of hypothalamic orexin neurons to the hippocampal CA1 region.

These findings demonstrate that blue light exposure activated the orexin‐hippocampus pathway and increases the activation of orexin neurons in the Lateral Hypothalamic Area. However, the expression of Orexin‐A was decreased in the hippocampus. We therefore speculate that the decrease in Orexin‐A expression in the hippocampus is induced by blue light exposure, whereas the heightened activation of orexin neurons in the hypothalamus may represent a compensatory response to alleviate blue light‐induced damage. Therefore, the orexin‐hippocampus pathway may play a protective role in counteracting cognitive impairment following blue light exposure.

### 3.5 The Orexin‐Hippocampus Pathway Improved the Exploratory Behavior, Learning, and Memory of Mice After Exposure to Blue Light

To verify whether the orexin–hippocampus pathway contributes to alleviating the cognitive and synaptic plasticity impairments induced by blue light exposure, we employed chemogenetic techniques in orexin‐Cre mice. First, to exclude the influence of orexin‐Cre on the experimental results, we initially examined the behavior of orexin‐Cre mice and wild‐type mice. A comparison of the behavior of wild‐type mice and orexin‐Cre mice in the open‐field (*n* = 6, 3.26% ± 3.04%, *p* = 0.31) (Figure S1A,B) and novel object recognition test (*n* = 6, 0.07% ± 0.05%, *p* = 0.05) (Figure S1C,D) revealed no significant difference in behavior between the two groups of mice. Further details are provided in Figure S1.

**FIGURE 6:**
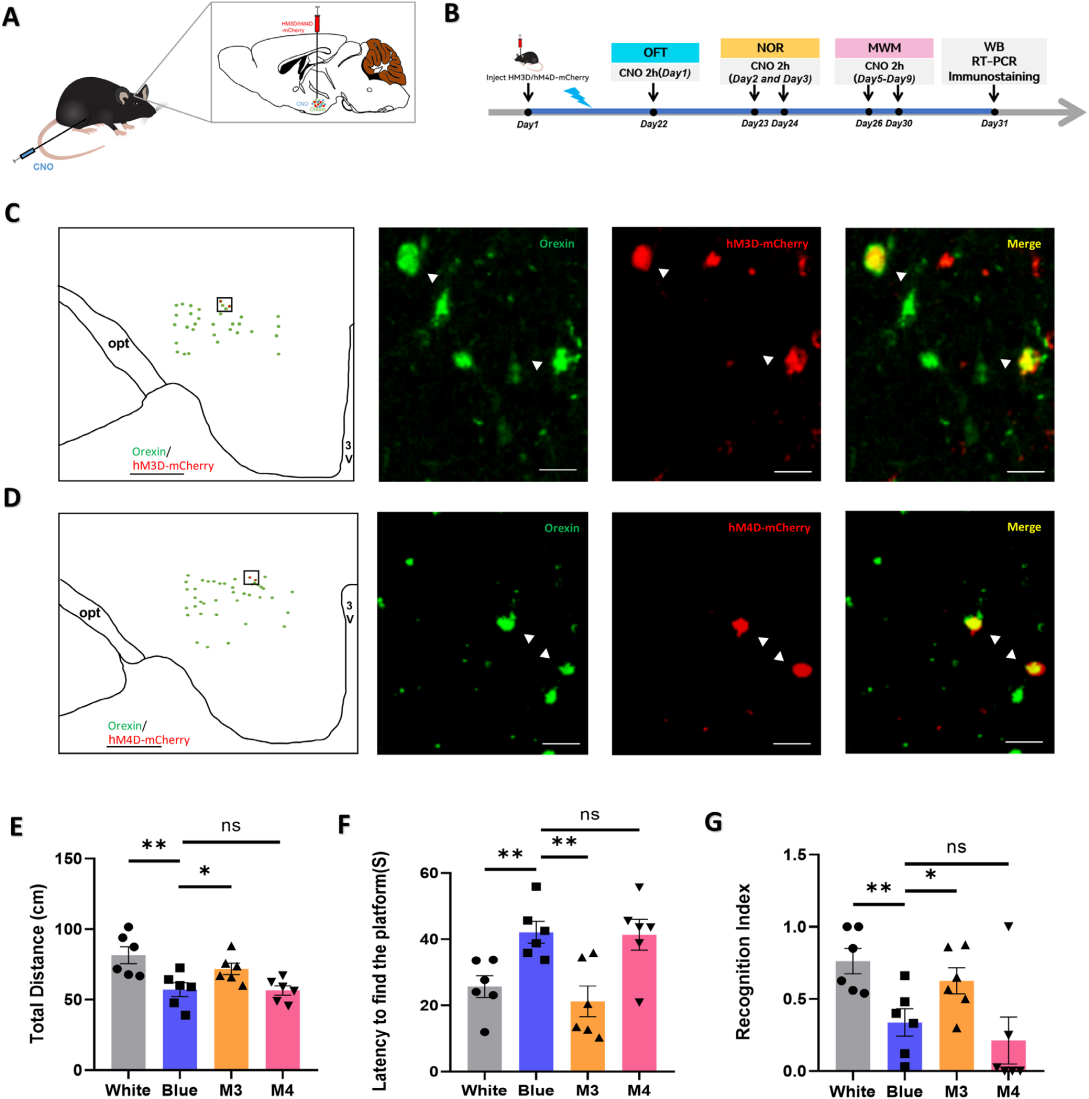
Regulation of the hypothalamic orexin‐hippocampus pathway by chemogenetics changes cognitive function. (A) Schematic diagram of hypothalamic virus injection and intraperitoneal clozapine N‐oxide (CNO) injection. (B) Time course diagram. (C, D) Images of the expression of hM3D‐mCherry (C) and hM4D‐mCherry (D) in the hypothalamus of orexin‐Cre mice, with squared areas enlarged in the images on the right (*n* = 6 mice per group). 3 V, 3rd ventricle; opt, optic tract. Scale bars: Left images 500 μm, right images 100 μm. (E) The blue light‐exposed mice traveled a longer total distance in the open‐field following activation of the M3 virus (*n* = 6 mice per group, *p* = 0.0474) but showed nonsignificant inhibition of the M4 virus (*n* = 6 mice per group). (F) The blue light‐exposed mice showed a decreased latency to find the platform in the Morris water maze following the activation of the M3 virus (*n* = 6 mice per group, *p* = 0.004) but did not significantly inhibit with M4 virus (*n* = 6 mice per group). (G) The blue light‐exposed mice spent more time on the novel object after activation of the M3 virus (*n* = 6 mice per group, *p* = 0.03) but did not significantly inhibit with M4 virus (*n* = 6 mice per group). The data are presented as the mean ± standard errors of the means; *p < 0.05, **p < 0.01, ns: Nonsignificant. *T*‐tests were performed.

Subsequently, rAAV‐Ef1α‐DIO‐hM3D (Gq)/hM4D (Gi)‐mCherry‐WPRES virus was injected into orexin‐Cre mice to induce orexin neurons expression of hM3Dq/hM4Di. Injection of CNO after 21 days of light exposure activated or inhibited the orexin–hippocampus pathway in orexin‐Cre mice (Figure 6A,B). Immunofluorescence staining showed that the hM3D (Gq)‐mCherry virus (Figure 6C) and hM4D (Gi)‐mCherry virus (Figure 6D) bound specifically to orexin neurons in the hypothalamus of orexin‐Cre mice.

Building upon this foundation, we noted a shift in the learning and memory capabilities related to the spatial position and direction (spatial direction) of mice. The detailed results are outlined below:

#### 3.5.1 Open‐Field Test Results

In comparison to the blue light exposure mice, the M3 mice exhibited a significant increase in the total distance covered within the open‐field area (*n* = 6, 13.37 ± 5.991, *p* = 0.0474) (Figure 6E). Conversely, the M4 mice showed no significant difference in entering the open‐field area but displayed a downward trend (Figure 6E). These findings suggested that the autonomous movement and exploration abilities of young mice were altered following the regulation of this pathway.

#### 3.5.2 Morris Water Maze Test Results

In contrast to the mice in the blue light group, the latency to find the platform decreased in the M3 virus injection group (*n* = 6, 20.87% ± 5.68%, *p* = 0.004) (Figure 6F), while no significant change was observed in the M4 group (Figure 6F). This suggested activation of the orexin–hippocampus pathway could enhance the learning and memory abilities related to the spatial position and orientation of blue light‐exposed mice.

#### 3.5.3 Novel Object Recognition Test Results

The findings in this section revealed that the RI of the M3 group showed an increase compared to the blue light‐exposed group (*n* = 6, 0.28% ± 0.12%, *p* = 0.03) (Figure 6G), indicating an enhancement in the learning ability of mice. Conversely, the RI of M4 is decreased (although not statistically significant) (Figure 6G), suggesting a potential impairment in their learning ability.

Based on the above results, we thought enhanced activation of the orexin‐hippocampus pathway alleviated cognitive impairment in mice after blue light exposure.

### 3.6 The Orexin–Hippocampus Pathway Improved the Hippocampal Synaptic Plasticity of Mice After Exposure to Blue Light

**FIGURE 7:**
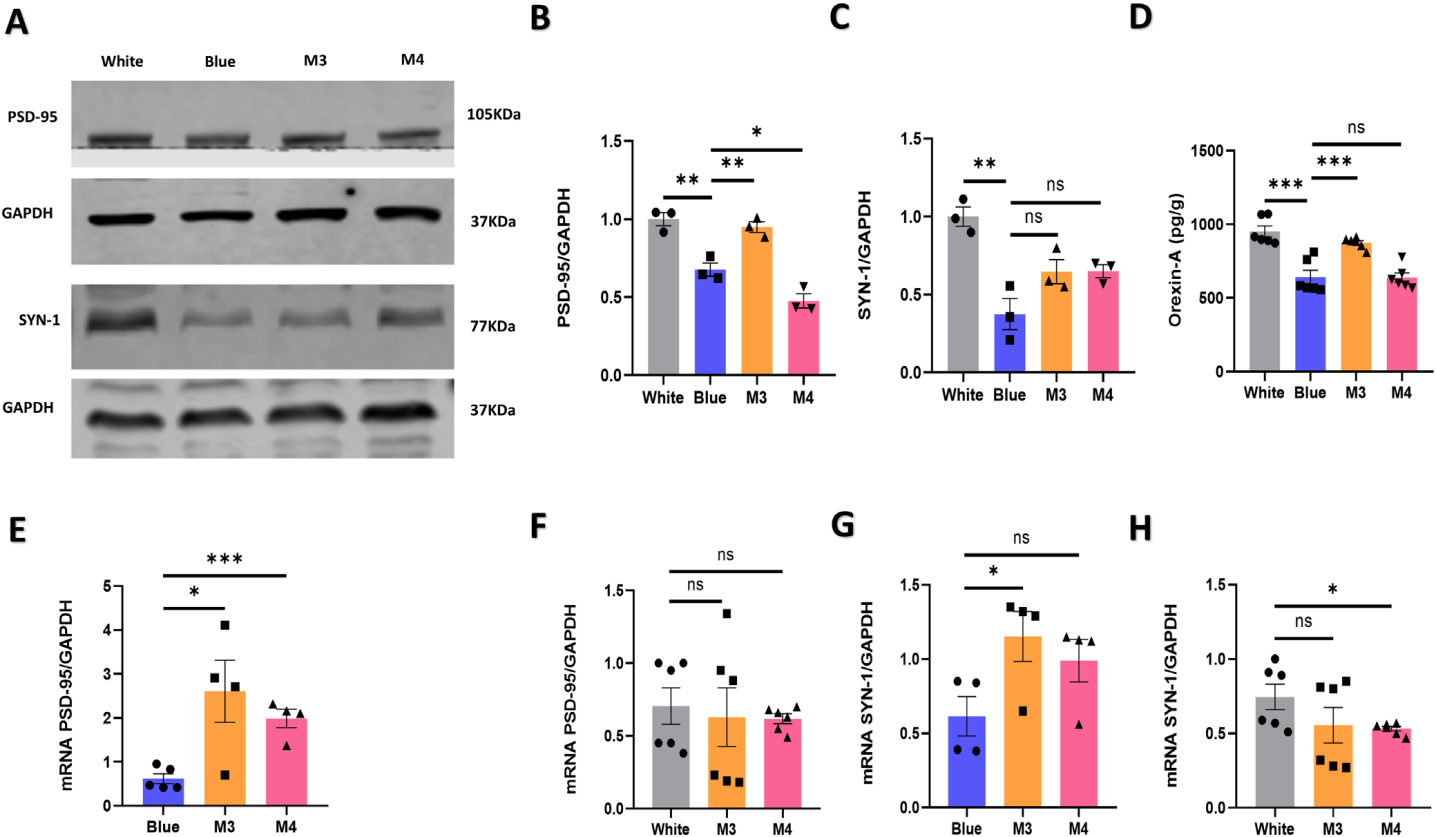
Regulation of the hypothalamic orexin‐hippocampus pathway by chemogenetics changes the expression of PSD95, SYN‐1, and Orexin‐A. (A) Western blotting analysis revealed the expression of PSD‐95 and SYN‐1 in the hippocampus. (B) Quantitative analysis showed that the expression level of PSD‐95 increased in the M3 group (*p* = 0.008) and decreased in the M4 group (*p* = 0.03). Blue, *n* = 3; M3, *n* = 3; M4, *n* = 3. (C) Quantitative analysis showed that the expression level of SYN‐1 is non‐significant in the M3 and M4 groups. Blue, *n* = 3; M3, *n* = 3; M4, *n* = 3. (D) Quantitative analysis showed that the expression level of Orexin‐A is increased in the M3 group (*p* = 0.0008) and is non‐significant in the M4 group. Blue, *n* = 6; M3, *n* = 6; M4, *n* = 6. (E) Quantitative analysis showed that the mRNA level of PSD‐95 is increased in the M3 group (*p* = 0.0164) and M4 group (*p* = 0.0005). Blue, *n* = 5; M3, *n* = 4; M4, *n* = 4. (F) The quantitative analysis showed that the mRNA level of PSD‐95 is non‐significant in the M3 group and M4 group. White, *n* = 6; M3, *n* = 6; M4, *n* = 6. (G) Quantitative analysis showed that the mRNA level of SYN‐1 is increased in the M3 group (*p* = 0.0275) and is non‐significant in the M4 group. Blue, *n* = 4; M3, *n* = 4; M4, *n* = 4. (H) Quantitative analysis showed that the mRNA level of SYN‐1 is non‐significant in the M3 group and is decreased in M4 group (*p* = 0.035). White, *n* = 6; M3, *n* = 6; M4, *n* = 6. The data are presented as the mean ± standard errors of the means; *p < 0.05, **p < 0.01, ***p < 0.001, ns: Nonsignificant. *T*‐tests were performed.

Similarly, the activated virus M3 was administered into the hypothalamic region of orexin‐Cre mice, followed by 21 days of blue light exposure and subsequent specific activation with CNO. Western blotting was employed to assess the levels of the synaptic plasticity‐associated protein factor PSD‐95 in the hippocampus. In comparison to the blue‐light‐exposed mice, PSD‐95 showed an up‐regulation (*n* = 3, 0.27% ± 0.06%, *p* = 0.008) (Figure 7A,B), and another factor, SYN‐1, displayed an upward trend, though without statistical significance (Figure 7A,C). Upon injection of the inhibitory virus M4, PSD‐95 was down‐regulated compared to blue‐light‐exposed mice (*n* = 3, 0.21% ± 0.06%, *p* = 0.03) (Figure 7A,B), and a similar downward trend, although not statistically significant, was observed for SYN‐1 (Figure 7A–C). We also assessed the levels of Orexin‐A in the hippocampus by Elisa and observed that the M3 activation group exhibited an increase in neurotransmitter A content (*n* = 6, 233.3% ± 49.01%, *p* = 0.0008) (Figure 7D), whereas the M4 inhibition group showed no statistically significant change (Figure 7D). Additional details are provided in Table S1.

Building upon these findings, we used the RT‐qPCR technique to investigate the changes at the gene level. *Psd‐95* exhibited up‐regulation after the injection of the activated M3 virus (*n* = 4, 1.99% ± 0.63%, *p* = 0.0164) and inhibitory M4 virus (*n* = 4, 1.370% ± 0.23%, *p* = 0.0005) (Figure 7E,F). Similarly, *Syn‐1* showed a comparable pattern to *PSD‐95*, with up‐regulation after activated virus injection (*n* = 4, 0.54% ± 0.21%, *p* = 0.0459) (Figure 7G,H).

## 4 Discussion

In this study, we innovatively demonstrated that: (i) prolonged exposure to blue light impaired cognitive behaviors and synaptic plasticity in mice. (ii) The activity of orexin‐hippocampus projections was significantly increased, and levels of orexin‐A and several factors related to synaptic plasticity in the hippocampus were significantly decreased following 21 days of blue light exposure. (iii) By specifically activating the orexin‐hippocampus projections using chemogenetics, cognitive impairments were alleviated, and synaptic plasticity was improved in those blue light‐exposed mice.

Given the 21 days duration of blue light exposure, which represents a relatively prolonged stress period, we speculate that the mice had sufficient time to initiate compensatory biological responses to counteract the damage. One such mechanism may involve the upregulation of orexin neurons activity and increased signaling along the orexin–hippocampus pathway, thereby helping to resist blue light–induced impairment. Thus, we propose that the heightened activation of orexin neurons and their hippocampal projections constitutes a protective response to blue light exposure, and that chemogenetic activation of this pathway can further facilitate learning and memory.

Exposure to electronic screens significantly influences the brain development and learning‐memory functions of preschool children [27]. Previous studies indicate that 21 days of exposure to blue light diminishes learning and memory in mice [28], and that early developmental light exposure impacts learning and memory storage in adults [29]. However, the specific mechanisms underlying the alleviation of blue light‐induced cognitive impairment remain unclear. Blue light‐induced cognitive impairment involves multiple biological mechanisms. Current evidence indicates that blue light disrupts melatonin secretion and circadian rhythms via the intrinsically photosensitive retinal ganglion cells (ipRGCs)‐suprachiasmatic nucleus (SCN) pathway, thereby contributing to sleep disturbances and cognitive decline [30, 31]. Moreover, ipRGCs may influence the prefrontal cortex (executive function) and hippocampal activity through retinothalamocortical projections [32, 33]. Blue light also elevates reactive oxygen species (ROS) in neural tissues, triggering neuronal damage and neuroinflammation that compromises hippocampal function [34].

Besides, the effects of blue light exposure on various brain regions and neurotransmitter systems demonstrate significant dependence on wavelength, duration, and circadian rhythm. Research indicates that short‐wavelength blue light (460–480 nm) can enhance prefrontal cortex (PFC) activation and improve cognitive function through non‐image‐forming visual pathways such as intrinsically photosensitive retinal ganglion cells (ipRGCs), while chronic nighttime exposure may impair its resting‐state functional connectivity [35, 36]. The impact of blue light on the amygdala exhibits a bidirectional pattern: acute exposure enhances its response to emotional stimuli [37], whereas prolonged nighttime exposure may reduce brain‐derived neurotrophic factor (BDNF) expression in the amygdala and induce anxiety‐like behaviors [38]. In the dopamine system, blue light exposure reduces dopamine release by inhibiting the activity of dopaminergic neurons in the retina, thereby affecting mood regulation and potentially contributing to the development of mood disorders such as depression and anxiety [39, 40]. Blue light exposure also suppresses melatonin secretion, with melatonin and dopamine demonstrating mutual inhibition and antagonism in retinal pathways [41, 42]. Although blue light therapy can alleviate depressive symptoms by increasing prefrontal serotonin (5‐HT) release, nighttime exposure inhibits melatonin secretion and indirectly disrupts serotonin metabolism [43, 44]. In our study, the blue light exposure decreased the expression of orexin‐A, PSD‐95, and SYN‐1, leading to reduced dendritic complexity and spine density, impaired synaptic plasticity in the hippocampus, and cognitive deficits.

Studies have indicated that blue light exerts significant effects on circadian rhythms and sleep through the melanopsin‐mediated ipRGCs‐SCN pathway. Short‐wavelength blue light (460–480 nm) effectively resets circadian phase, while nocturnal exposure suppresses melatonin secretion, prolongs sleep latency, and reduces slow‐wave sleep. The study exposed mice to 460‐nm blue light (1000 lx intensity) for 12–16 h daily over 4 weeks. Compared with dark controls, the findings revealed that blue light regulates glucose metabolism via a retina–hypothalamus–brown adipose tissue axis [45]. Concurrently, blue light directly activates hypothalamic orexin neurons, influencing sleep–wake balance via a dual regulatory mechanism (promoting wakefulness while inhibiting sleep centers). The study achieved precise control of wakefulness in transgenic mice by specifically stimulating orexin neurons with 470‐nm blue light (1.5–2.0 mW/mm^2^ intensity), delivered in 10 Hz pulses with 30s ON/30s OFF cycles [46]. In contrast, our experimental findings showed that although blue light activated orexin neurons, no behavioral signs of drowsiness were observed during or after exposure. This discrepancy may be attributed to methodological differences in the light exposure models. In our study, mice were exposed to either white light (150 lx) or dim blue light (5 lx, 480 nm, 8000 K) under a 12:12‐h light–dark cycle (light phase from 07:00 to 19:00) for 21 consecutive days. Light exposure was administered using LED‐illuminated cabinets, with light intensity verified using a lux meter at Zeitgeber Time, during which the rodents were naturally awake.

This study demonstrated that blue light exposure impairs cognitive function in mice and activates the neural pathways from hypothalamic orexin neurons to the hippocampus. Furthermore, artificial regulation of these pathways leads to improvement in learning, memory, and prominent synaptic plasticity.

Our experiment revealed that mice with cognitive impairments caused by blue light exposure, the level of Orexin‐A in their hippocampus also decreased. These findings align with earlier studies showing that increased orexin levels regulate spatial learning and memory [47]. Orexin neurons are primarily localized in a specific subregion of the tuberal region of the hypothalamus, and their axonal projections are widespread throughout the brain, including the hippocampus, a crucial nucleus in the regulation of learning and memory [48, 49]. Several investigations have indicated that orexin can influence spatial learning and memory by modifying the connection structure and function of the hippocampus. As the central hub for learning and memory, the hippocampus undergoes synaptic plasticity induced by orexin receptors, which regulate the acetylcholine, glutamatergic, γ‐aminobutyric acid (GABA), and adrenergic systems [50]. Furthermore, orexin deficiency produces context‐dependent effects on learning and memory, with impairments primarily emerging under cognitively demanding conditions [51]. Although orexin knockout (KO) mice generally show preserved performance in standard spatial and fear memory tasks [52, 53], significant deficits become apparent when the cognitive load increases or when animals experience sleep deprivation [54]. The stress‐induced impairments also reveal orexin's critical role in maintaining cognitive resilience [55]. Notably, orexin KO particularly disrupts cue‐dependent fear memory [51] and tasks requiring sustained attention.

In addition to orexin neurons, a substantial population of c‐FOS^+^ non‐orexin neurons was also observed in the hypothalamus (Figure 5G), indicating the involvement of other hypothalamic subpopulations in the response to blue light. Within this specific region of the hypothalamus, potential neuronal populations may include glutamatergic, GABAergic, corticotropin‐releasing hormone (CRH), and melanin‐concentrating hormone (MCH) neurons [56, 57]. Studies conducted on anesthetized mice demonstrated that blue light exposure significantly increased the total number of FOS‐expressing neurons in the lateral hypothalamic area [58]. Blue light deprivation resulted in lower CRH in the hypothalamus of gerbils [59]. MCH neurons, traditionally recognized for their primary role in regulating feeding and sleep behaviors, have now been substantiated to be associated with cognitive functions [60]. GABAergic neurons are extensively distributed throughout the hypothalamus, where they play crucial roles in diverse physiological functions and neuromodulatory processes [61], and interact with both orexin and MCH neurons [62, 63]. Although this study did not identify the specific cell types of these non‐orexin FOS^+^ neurons, based on the above reports, we propose that GABAergic, CRH, and MCH neurons within the hypothalamus may also participate in the responses induced by blue light exposure. Therefore, further investigations are needed to precisely determine the mechanisms by which these neuronal populations participate in the blue light exposure response.

Contrary to our expectations, chemogenetic inhibition of the hypothalamic orexin‐hippocampus pathway did not further inhibit learning, memory, or cognitive functions in mice exposed to blue light. One plausible explanation is that various orexin neurons may project to other brain regions, such as the nucleus accumbens [64], the medial prefrontal cortex [65], and the basal ganglia [66], exerting a compensatory effect on learning and memory when the orexin neurons terminals in the hippocampus are inhibited. However, this hypothesis requires additional experimental validation. Another consideration is the possibility that hM4D (Gi)‐mCherry may exhibit reduced efficacy when acting on presynaptic terminals, necessitating more effective inhibition experiments for confirmation [67]. Besides, previous research also indicates that chemogenetic inhibition of orexin neurons does not directly affect learning and memory abilities [68]. Additionally, the effects of orexin neurons on memory may depend on their projections to the locus coeruleus (noradrenergic) or prefrontal cortex rather than direct actions on the hippocampus [69]. Hence, the orexin system primarily supports cognitive function indirectly by sustaining wakefulness, rather than directly regulating memory encoding or storage processes in the hippocampus. This may explain why inhibiting orexin neurons does not impair memory function.

## 5 Conclusion

In summary, this study demonstrated that 21 days of blue light exposure significantly impaired cognitive behaviors in mice, as evidenced by open‐field, novel object recognition, and Morris water maze tests. Electrophysiological recordings and Golgi staining confirmed that prolonged blue light exposure impaired hippocampal synaptic plasticity in mice. Biochemical analyses revealed downregulation of Orexin‐A and synaptic plasticity‐related factors PSD‐95 and SYN‐1 at both protein and gene levels in the hippocampus following blue light exposure. Retrograde tracing combined with immunofluorescence staining identified an orexin‐hippocampus pathway formed by hypothalamic orexin neuron projections to the hippocampal CA1 region. Chemogenetic activation of this pathway notably improved cognitive function and upregulated the expression of Orexin‐A, PSD‐95, and SYN‐1 in mice subjected to blue light exposure. We speculate that the hypothalamic orexin‐hippocampus pathway may exhibit feedback‐enhanced activity to alleviate synaptic plasticity and cognitive impairments induced by blue light exposure. Although this experimental evaluation is limited to short‐term data following blue light exposure and lacks long‐term assessment, the novel findings regarding the role of orexin‐hippocampus projections offer new ideas for subsequent long‐term effects research and clinical interventions for blue light exposure‐induced impairments.

## Author Contributions

J.N. and D.D. conceived and designed the experiments, interpreted the data, and critically revised the manuscript. Z.F., Qin.L., Z.H., B.Y., T.M., and Y.H. performed the experiments and acquired the data. Z.F., J.N., Qin.L., Qi.L., and X.C. analyzed the data. Z.F. and Qin.L. drafted the manuscript. All authors read and approved the final version of the manuscript.

## Ethics Statement

The animal study protocol was reviewed and approved by the Laboratory Animal Ethics and Welfare Committee of the Laboratory Animal Center, Ningxia Medical University's.

## Conflicts of Interest

The authors declare no conflicts of interest.

## Supporting information


**Table S1:** cns70551‐sup‐0001‐TableS1.tif.

**Figure S1:** cns70551‐sup‐0002‐FigureS1.tif.

## Data Availability

The data generated or analyzed during this study are included in this published article.
