# Supplementary figures and images for: Hypothalamic Orexin Projections to the Hippocampal CA1 Region Alleviate Cognitive and Synaptic Plasticity Impairments Induced by Blue Light Exposure
Source: CNS Neurosci Ther. 2025 Aug 17;31(8):e70551. doi: 10.1111/cns.70551 (PMC12358687; doi:10.1111/cns.70551)

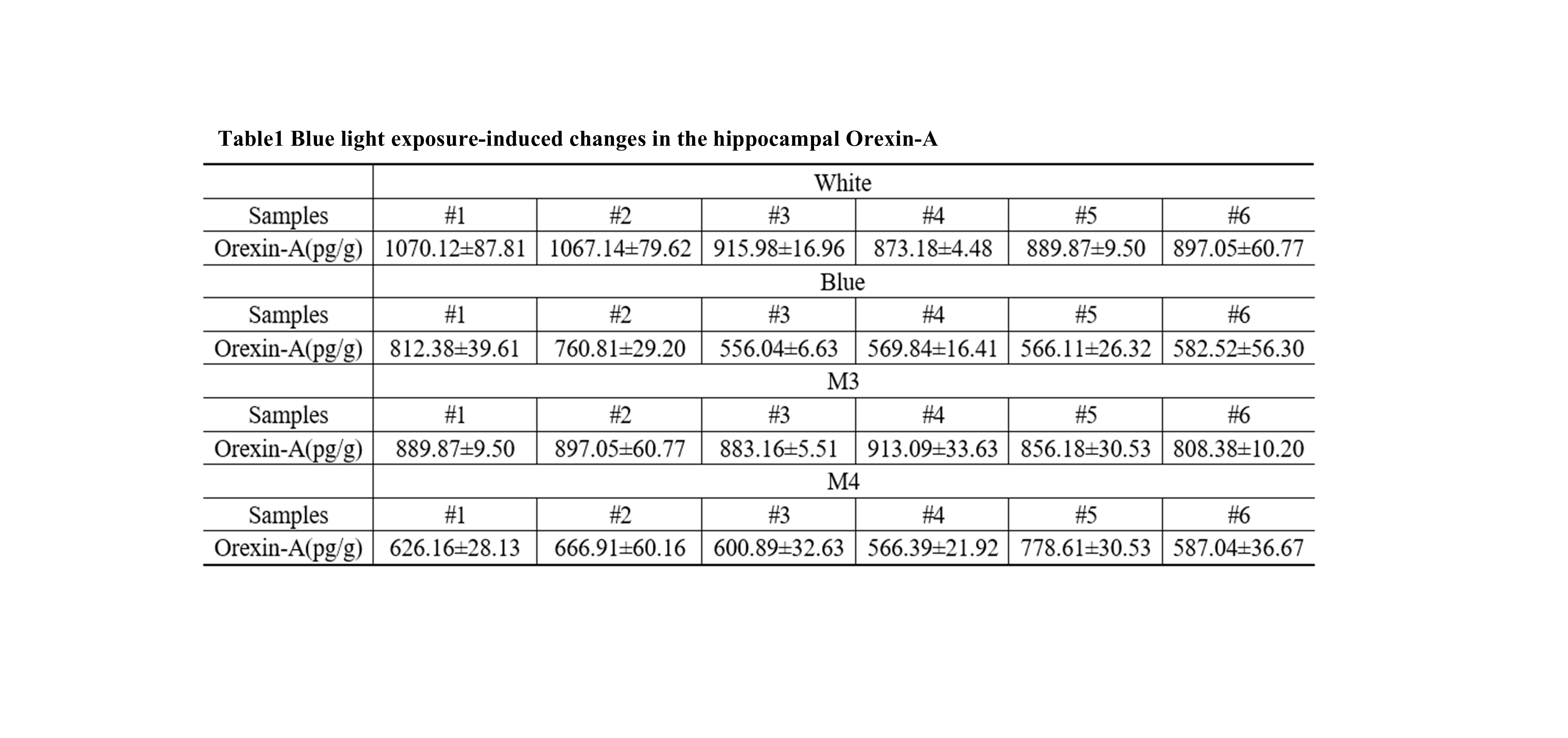

Supplement: Supplementary file 1 — Table S1: cns70551‐sup‐0001‐TableS1.tif. [file CNS-31-e70551-s002.tif]

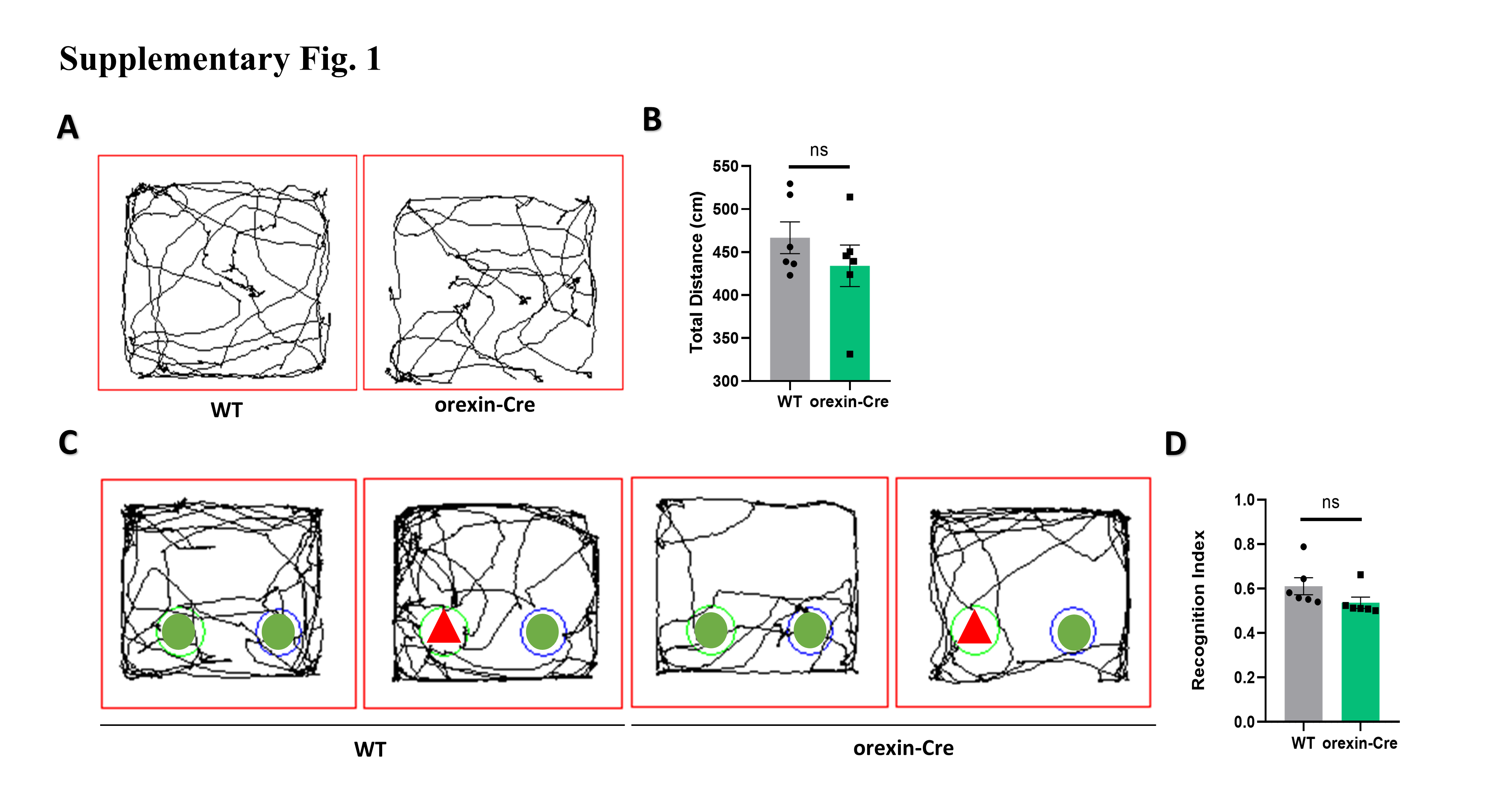

Supplement: Supplementary file 2 — Figure S1: cns70551‐sup‐0002‐FigureS1.tif. [file CNS-31-e70551-s001.tif]
